# Cervical length versus vaginal PH in the second trimester as preterm birth predictor

**DOI:** 10.12669/pjms.312.6310

**Published:** 2015

**Authors:** Fatemeh Foroozanfard, Zohreh Tabasi, Elaheh Mesdaghinia, Mojtaba Sehat, Mahdian Mehrdad

**Affiliations:** 1 Fatemeh Foroozanfard, Department of Gynaecology and Obstetrics, Kashan University of Medical Sciences, Kashan, Iran; 2 Zohreh Tabassi, Department of Gynaecology and Obstetrics, Kashan University of Medical Sciences, Kashan, Iran; 3 Elaheh Mesdaghinia, Department of Gynaecology and Obstetrics, Kashan University of Medical Sciences, Kashan, Iran; 4 Mojtaba Sehat, Department of Community Medicine, Kashan University of Medical Sciences, Kashan, Iran; 5 Mehrdad Mahdian, Trauma Research Center, Kashan University of Medical Sciences, Kashan, Iran

**Keywords:** Bacterial vaginosis, Cervical length measurement, Preterm birth, Second trimester

## Abstract

**Objective:**

To evaluate diagnostic value of vaginal pH and cervical length measurement in the second trimester of pregnancy as a preterm labor (PTL) predictor.

**Methods:**

During a prospective cohort study 438 uncomplicated singleton pregnant women between 18 and 24 weeks of gestation were assessed regarding vaginal PH and cervical length. Vaginal pH was measured using Ph-indicator strips and cervical length was determined using transvaginal ultrasound. The cut-off values for vaginal PH and cervical length were defined as 5 and <30 mm respectively.

**Results:**

Vaginal pH of 5 and above was found in 162/438 women (37%) while cervical length <30mm was found in 38/438 (8.7%). The incidence of PTL < 37 weeks was 87/438 (19.9%) while the incidence of early (PTL <34 weeks) was 51/438 (11.6%). Predictive value of higher vaginal PH was significantly more (31%) than vaginal PH<5 (13%) in predicting PTL. As a result, alkaline vaginal PH significantly increases the odds of preterm labor (OR=3.06). Shortened cervical length is better predictor of PTL than higher vaginal PH with positive predictive value of 71% and negative predictive value of 85%. Cervical length less than 30 mm nearly 14-fold increases odds of preterm birth (OR=13.9).

**Conclusion:**

Compared to alkaline vaginal PH, shortened cervical length has better value to predict PTL overall. However, regarding early or late PTL, vaginal PH is more accurate to predict late PTL, while cervical length measurement is more appropriate to predict early PTL (<34 weeks).

## INTRODUCTION

In spite of advances achieved in perinatal care, prematurity has remained a major public health problem and is a leading cause of death and morbidity and imposes large costs to the health care system.^1^,^2^ Early detection of pregnant women at risk of premature labor (PTL) will help to reduce the occurrence of prematurity- related mortality and morbidity. Incidence of premature birth is approximately 9.6% of all birth globally.^3^ Cervical insufficiency and bacterial vaginosis are two items that recently known play an essential role in preterm delivery. They can be diagnosed using safe, simple and reliable methods. These problems are also potentially treatable.^4^-^6^ Bacterial vaginosis (BV) is a lower genital tract infection characterized by change in normal vaginal flora and replacement by vaginosis-associated anaerobic microorganisms. BV is a leading cause of many undesirable fetal outcomes including premature birth and premature rupture of fetal membranes.^7^,^8^ This problem leads to an increase in vaginal pH that may be diagnostic.^9^ Cervical insufficiency describes a functional weakness of the cervix usually associated with short cervical length, less than 3 centimeters.^10^ Transvaginal ultrasound assessment of the cervix has been used as a diagnostic measurement for prediction of preterm labor. Women with history of preterm labor, preterm premature rupture of membranes (PPROM), in utero diethylstilbestrol (DES) exposure, or collagen disorders (e.g., Ehlers-Danlos syndrome), cervical lacerations during labor and delivery and cervical injuries due to gynecologic procedures are at risk of premature labor.^11^,^12^ Transvaginal ultrasound screening, cervical length measurements and early intervention like cerclage prevent premature birth.^12^

Women who do not meet the mentioned criteria are categorized as low risk group and there is no evidence that cervical length measurement is useful in such cases for preterm birth prediction. However, the recent studies suggest that it is better to measure cervical length in women who are referred to ultrasound examination to rule out fetal anomalies,^10^ pregnancy dating, screening for abnormalities and monitoring of fetal growth. Since recent evidence suggests that more effective screening of premature birth can be provided by the sonographic measurement of cervical length at second trimester of pregnancy^13^-^15^ and on the other hand, some studies propose that prevention of PTL is possible by vaginal pH screening,^16^ this study was designed to compare the accuracy of a sign of bacterial vaginosis (increased vaginal PH) and a sign of cervical insufficiency (cervical length <3 cm) in predicting preterm labor.

## METHODS

This prospective cohort study was conducted on 438 pregnant women with uncomplicated singleton pregnancy between 18 and 24 weeks of gestation. Based on Sensitivity 75%, 1-SP= 19.9% (17) and regarding negligible error of 0.2 and confidence interval 95% and power of 90%, minimum sample size was calculated as 81 in each group of parturient (PTL and term labor).

All cases were recruited in the study as they came to perinatal care clinics of Kashan University of Medical Sciences and private offices for routine pregnancy follow up during October 2012- December 2013. Ethic committee of Kashan University of Medical Sciences approved the study protocol and informed written consent was received from all participants. In a pilot study it was found that among the all cases that were referred for delivery, 20 percent were from clinics and other referred from private offices. Therefore, in this study and during the sampling, this ratio was adhered.

The exclusion criteria were suspected chorioamnionitis (fever>38.5), vaginal bleeding, history of surgical procedure on the cervix, cervical cerclage, autoimmune disease, sexual intercourse or use of products that can affect vaginal pH over the past 24 hours, major congenital fetal anomalies that were diagnosed in the previous ultrasound exam or screening tests for the first and second trimester of pregnancy. Gestational age was determined using last menstrual period and a correction made based on ultrasound measurements performed at first trimester. Vaginal PH was measured to assess bacterial vaginosis using Ph-indicator strips (Merck KGaA, 64271 Damstadt, Germany) after speculum insertion in lithotomy position. No lubricants were used in order to possible interaction with the results. PH measurement was performed by trained midwives. The vaginal PH of 5 was considered as cut - off point and defined as elevated in our study population. Our cut- off point has been used in many other studies.^16^,^18^,^19^

After the measurement of vaginal pH, transvaginal ultrasound examination was performed using Medison Accuvix-xo ultrasound imaging system (Samsung, Korea) to measure the length of cervix. To increase the validity and reliability of the vaginal PH measurements, same standard tests were used in all clinics and private offices. All midwives involved in measuring vaginal PH were trained and after certification of their performance by two gynecologists participated in sample collection. Accuracy and reliability of PH measurement were checked during the study by evaluation of midwives performance. To avoid differences in the level of medical care in both groups of the study, it was tried that the patient, physician and the investigator remain blind to the vaginal PH and the cervical length until delivery time.

However, to prevent health problems in patients, related codes were given to the physician if needed. All patients were followed until delivery and gestational age was determined at delivery time. SPSS software version 17 was used for statistical analysis. After primary clearing and correction of missing data, variables were described using descriptive statistics. Risk of preterm delivery among the study population was calculated in general and in terms of pH and cervical length and predictive value for each item was reported. Chi-square test and t-test were used for comparing results. Regression modeling was used to adjust for the effect of confounders. P-value less than 0.05 was considered statistically significant.

## RESULTS

Four hundred and fifty six women met inclusion criteria during the study period who were enrolled the study. However, 18 cases were lost from follow up due to different reasons. Finally, vaginal PH and cervical length was measured at 18 to 24 weeks in 438 pregnant women. Mean (SD) maternal age was 26.79(4.55) years. Mean gestational age at enrollment was 19.3 weeks. Mean (SD) gestational age at delivery was 37.9(2.5) weeks. Minimum gestational age at delivery was 18 weeks and maximum was 41. The incidence of PTL < 37 weeks in our cohort was 87/438 (19.9%) while the incidence of early PTL <34 weeks was 51/438 (11.6%).

Our study showed alkaline vaginal PH and short cervical length (<30mm) significantly increases the odds of preterm labor (OR=3.06 and OR=13.9 respectively). Compared to vaginal PH, cervical length had a greater positive predictive value (PPV) and positive likelihood ratio to predict PTL (Table-I). In addition, compared to cervical length, vaginal PH was more accurate in predicting late (34-37 weeks) than the early (<34 weeks) PTL (Table II and III). Multivariate logistic regression showed that even after adjusting for effect of age and parity, there is a significant relationship between vaginal PH and cervical length and preterm delivery (P<0.0001) (Table-IV).

**Table-I:** Indices for Vaginal PH≥5 and Cervical Length <30mm as Preterm labor predictors.

Variable	Sen % (CI)	SP % (CI)	PPV % (CI)	NPV % (CI)	+LR(CI)
PH≥5	58.5(47.6-69.1)	68.4(63.2-73.2)	31.5(24.4-39.2)	87.0(82.4-90.7)	1.85(1.47-2.34)
Cervical length<30mm	31.0(21.5-41.9)	96.8(94.4-98.4)	71.1(54-84.6)	85.0(81.1-88.3)	9.90(5.12-19)
P Value	<0.0001	<0.0001	<0.0001	0.427	<0.0001

***Abbreviations:*** Sen,; Sensitivity, CI; Confidence Interval, SP; Specificity, PPV; Positive Predictive Value, NPV; Negative Predictive Value; +LR; Positive likelihood Ratio.

**Table-II:** Frequency of early versus late PTL regarding vaginal PH.

		Preterm Labor	Normal Delivery	Total	P Value	OR
		Early preterm <34 weeks	Late preterm 34-37 weeks						
PH<5	Number	23	13	240	276	<0.0001 Late: 3.82
Early:2.63	%	8.3%	4.7%	87.0%	100.0%
PH ≥5	Number	28	23	111	162
	%	17.3%	14.2%	68.5%	100.0%
Total	Number	51	36	351	438
	%	11.6%	8.2%	80.1%	100.0%		

**Table-III:** Frequency of early versus late PTL regarding cervical length.

Variable	B	S.E	Odds Ratio	95%CI	P
Vaginal PH	1.002	0.169	2.73	1.96 - 3.79	<0.001
Cervical Length	-0.116	0.32	0.89	0.84 - 0.95	<0.001
Parity	0.961	0.237	2.61	1.64 - 4.16	<0.001
Age	-0.28	0.039	0.92	0.90 – 1.05	0.475

**Table IV:** Parameters of multivariate logistic regression for predicting PTL.

		Preterm Labor		Normal Delivery	Total	P Value	OR
		Early preterm <34 weeks	Late preterm 34-37 weeks				
Cervical length <30	Number	17	10	11	38	<0.0001	Late=11.9
	%	44.7%	26.3%	28.9%	100.0%		Early=15.5
Cervical length ≥30	Number	34	26	340	400
	%	8.5%	6.5%	85.0%	100.0%
Total	Number	51	36	351	438

## DISCUSSION

This study showed a significant correlation between alkaline vaginal pH and shortened ultrasound cervical length measurement regarding the prediction of PTL. In fact, Preterm labor in vaginal pH higher than 5 was seen to be three times more than the vaginal pH below 5 (OR=3.06) and also it is concluded that cervical length less than 30 mm almost 14-fold increases odds of preterm birth (OR=13.9). Series of studies were performed regarding the correlation of vaginal PH, cervical length and preterm labor.^20^-^26^ Regarding bacterial vaginosis and alkaline vaginal PH, the findings of the current study is almost consistent with those of Lim KH et al. who found, that women who were referred for preterm labor had approximately doubled chance of being diagnosed with bacterial vaginosis compared to control.^20^

Kumar S et al. conducted a prospective trial and recruited 120 women in their study, 60 women in spontaneous preterm labor with or without rupture of membranes were compared to 60 women in spontaneous labor at term pregnancy with or without rupture of membranes regarding bacterial vaginosis. PH testing of vaginal discharge and vaginal smear were used for bacterial vaginosis diagnosis. They concluded that attributive risk of preterm labor in women with bacterial vaginosis was 5.0476 (CI 95% 1.9677 to 12.952).^21^ Sendag F et al. via a prospective study not only found that there is a significant correlation between vaginal PH<5 and increase risk of preterm delivery (that supports our results), but also found simultaneously, there is a significant correlation between an elevated vaginal pH (> 5.0) and a shortened cervical length (r = -0.59, p < 0.001).^22^

Generally, our results further support the idea of almost all of mentioned studies. However, very little was found in the literature on the question of effect of vaginal PH on premature labor regarding early and late separately.^16^ Since based on our findings odds ratio of early and late PTL were 2.63 and 3.82 respectively, it is observed that BV increases the risk of late PTL more than early one. Our finding is comparable with the results of Pires CR et al and Thomas S et al. regarding correlation of cervical length and preterm labor and negligible differences in results may be explained by the differences in their cut- off point for short cervical length (less than 25 mm rather than our cut-off <30mm) and number of cases.^23^,^24^ However, Matijevic R et al. during a prospective cohort study to evaluate of diagnostic value of vaginal PH and cervical length in prediction of PTL concluded both measures have significant predictive value for PTL and between two criteria, elevated vaginal pH has better accuracy.^16^

These results differ from our findings regarding accuracy of vaginal PH and cervical length, since we concluded that short cervix is more valuable than vaginal pH as a PTL predictor. A possible explanation for this might be that they used cervical length of <25 mm as their cut-off point. In some studies even shorter cervical length (<15 mm) were considered as a risk factor for PTL and also showed it has a relatively limited value as a single measure for predicting PTL.^25^,^26^ This inconsistency with our results may be due to a number of reasons like different methodology and analysis.

In conclusion, based on our study, either cervical length measurements or vaginal pH assessment can be considered as preterm labor predictors in low risk population and cervical length measurement has better diagnostic value overall. However, vaginal PH appeared to be more accurate in the prediction of late PTL, while cervical length measurement was more appropriate to predict early PTL<34 weeks.
